# Characterization of Molecular Determinants of the Conformational Stability of Macrophage Migration Inhibitory Factor: Leucine 46 Hydrophobic Pocket

**DOI:** 10.1371/journal.pone.0045024

**Published:** 2012-09-21

**Authors:** Farah El-Turk, Bruno Fauvet, Amer Ashrafi, Hajer Ouertatani-Sakouhi, Min-Kyu Cho, Marilisa Neri, Michele Cascella, Ursula Rothlisberger, Florence Pojer, Markus Zweckstetter, Hilal Lashuel

**Affiliations:** 1 Laboratory of Molecular and Chemical Biology of Neurodegeneration, Department of Life Sciences, Swiss Federal Institute of Technology, Lausanne, Switzerland; 2 Department of NMR-Based Structural Biology, Max Planck Institute for Biophysical Chemistry, Gottingen, Germany; 3 Laboratory of Computational Chemistry and Biochemistry, Department of Chemistry, Swiss Federal Institute of Technology, Lausanne, Switzerland; 4 Global Health Institute GHI, Department of Life Sciences, Swiss Federal Institute of Technology, Lausanne, Switzerland; Institute of Enzymology of the Hungarian Academy of Science, Hungary

## Abstract

Macrophage Migration Inhibitory Factor (MIF) is a key mediator of inflammatory responses and innate immunity and has been implicated in the pathogenesis of several inflammatory and autoimmune diseases. The oligomerization of MIF, more specifically trimer formation, is essential for its keto-enol tautomerase activity and probably mediates several of its interactions and biological activities, including its binding to its receptor CD74 and activation of certain signaling pathways. Therefore, understanding the molecular factors governing the oligomerization of MIF and the role of quaternary structure in modulating its structural stability and multifunctional properties is crucial for understanding the function of MIF in health and disease. Herein, we describe highly conserved intersubunit interactions involving the hydrophobic packing of the side chain of Leu46 onto the β-strand β3 of one monomer within a hydrophobic pocket from the adjacent monomer constituted by residues Arg11, Val14, Phe18, Leu19, Val39, His40, Val41, Val42, and Pro43. To elucidate the structural significance of these intersubunit interactions and their relative contribution to MIF’s trimerization, structural stability and catalytic activity, we generated three point mutations where Leu46 was replaced by glycine (L46G), alanine (L46A) and phenylalanine (L46F), and their structural properties, stability, oligomerization state, and catalytic activity were characterized using a battery of biophysical methods and X-ray crystallography. Our findings provide new insights into the role of the Leu46 hydrophobic pocket in stabilizing the conformational state of MIF in solution. Disrupting the Leu46 hydrophobic interaction perturbs the secondary and tertiary structure of the protein but has no effect on its oligomerization state.

## Introduction

Macrophage Migration Inhibitory Factor (MIF) is a ubiquitous multifunctional protein and a key player in the inflammatory response and innate immunity. MIF was first identified in the 1960s as a T-cell cytokine involved in the delayed type hypersensitivity and several macrophage functions, including secretion and production of proinflammatory cytokines [1], [2]. During the last two decades MIF has been shown to be involved in a wide range of cellular processes, *e.g.* transcriptional regulation of inflammatory gene products [3], cell cycle control [4], [5], modulation of cell proliferation and differentiation [6], regulating glucocorticoïd activity [7], inactivation of p53 tumor suppressor factor [8] and signal transduction, and emerged as an important player in the molecular mechanisms underlying the pathogenesis of several inflammatory autoimmune diseases including arthritis [9], [10], [11], multiple sclerosis [12], [13], diabetes [14], sepsis [15], [16], [17], atherosclerosis [18] and oncogenesis [19], [20], [21], [22], [23], [24], [25]. The role of MIF in these diseases has been confirmed in several animal models using genetic, immunological and pharmacological approaches.

Unlike other cytokines, MIF also functions as an enzyme, and exhibits hormone-like activities [26], [27], [28]. MIF has two enzymatic activities: an evolutionarily well conserved keto-enol tautomerase activity [29], [30] and a thiol-protein oxido-reductase activity that is mediated by the C_56_ALC_59_ motif [31], [32]. However, the physiological relevance of these activities and their role in regulating the function of MIF in health and disease remain controversial [33], [34]; the physiological substrates for both catalytic activities are yet to be discovered.

**Figure 1:**
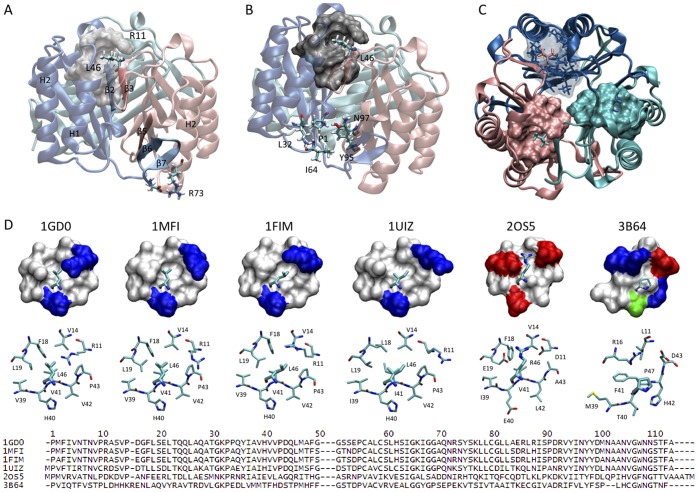
The Leu46 hydrophobic pocket is highly conserved across MIF species. The three MIF monomers are represented as cartoons and are colored in pink, cyan and blue. (A) Side view of the trimer illustrating the different intersubunit interactions. Two main regions within each monomer were shown to be responsible for the protein trimerization: each subunit interacts with one neighbouring monomer through tight interactions involving the inner β-strand (β3) and with the other neighbouring monomer though the C-terminal β-hairpin (β6 and β7). The C-terminal β-hairpin comprises two major types of interactions: 1) intersubunit β-sheet, and 2) salt-bridge interactions. The β-strand β3 contributes to trimer stabilization through two types of contacts: 1) intersubunit β-sheet formation, and 2) hydrophobic interactions between the side chain of Leu46 localized on the β-strand β3 and a hydrophobic pocket from the adjacent monomer constituted by residues Arg11, Val14, Phe18, Leu19, Val39, His40, Val41, Val42, Pro43. (B) Side view of the trimer illustrating the distance between the Leu46 hydrophobic pocket and the enzymatic site. (C) Top view of huMIF showing the three hydrophobic pockets where Leu46 from adjacent monomers are packed. Each pocket is represented with the same color as the subunit it belongs to, Leu46 are represented as stick models licorice and colored with the same subunit color. Structural data according to Orita *et al.* [51], PDB code: 1GD0. (D) Hydrophobic pocket structure homology between different MIF species. Structural data were generated using the following PDB files: 1GD0 (human MIF, [51]); 1MFI (mouse MIF, [68]); 1FIM (rat MIF, [69]); 2OS5 (*Ancylostoma ceylanicum* MIF, [70]); 3B64 (*Leishmania* parasite MIF, [71]); 1UIZ (*Xenopus laevis* MIF, [72]). Amino acids are represented on the figure with one-letter codes.

X-ray structural studies have consistently shown that MIF exists as a homotrimer [35]. Data from size-exclusion chromatography [36], analytical ultracentrifugation [36], [37] and light scattering [36] are also consistent with the trimer as the predominant species in solution, although a number of reports suggest that MIF may populate a mixture of trimeric, dimeric and monomeric states at physiological concentrations [38], [39]. Each MIF monomer consists of 114 amino acids and is composed of two anti-parallel α-helices packed against a four-stranded β-sheet. The trimer is held together by a range of intersubunit interactions involving key residues from two primary regions within each monomer [36]; i) the inner β-strand β3 of each monomer (**Figure 1A**); ii) the C-terminal region of MIF, including the C-terminal β-hairpin comprising residues 105–114 (β6, β7), is involved in several intersubunit stabilizing interactions. Previous studies from our laboratory and others [36], [40], [41] have assessed the importance of the conformational properties of this region on the oligomerization and functional properties of huMIF. C-terminal deletions (110–114 or 105–114) or disruption of the conformational properties of this region, via insertion of a proline residue, result in loss of MIF’s enzymatic activity [36], [40], [41] and reduction in macrophage activating properties [41]. At the structural level, these mutations were shown to induce significant tertiary structure changes within the MIF trimer without altering its oligomerization state and receptor (CD74) binding properties [36].

**Figure 2:**
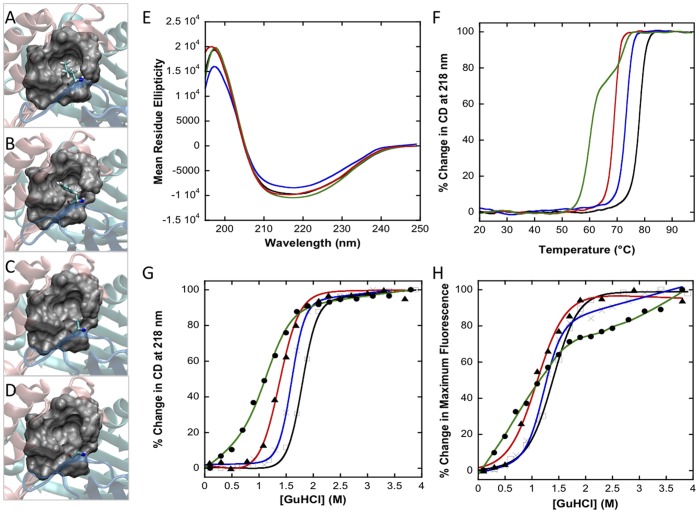
Disrupting the hydrophobic interactions via mutating Leu46 alters the structural stability of MIF. (A) Leu46 hydrophobic pocket of wt huMIF. VMD representations of the hydrophobic pocket, where Leu46 is mutated to a phenylalanine (L46F) (B), alanine (L46A) (C), or glycine (L46G) (D). (*E-H):* The three Leu46 mutants are structurally less stable than the wild type protein, but retain the same overall secondary structure. (E) Far-UV CD spectra of wt and Leu46 mutants. (F) Thermal denaturation of wt and Leu46 mutants (at 20 µM) followed by far-UV CD at 218 nm. (G) GdnHCl denaturation studies monitored by far-UV CD at 218 nm and fluorescence spectroscopy (H), excitation wavelength: 295 nm, protein concentration: 10 µM. All spectroscopic experiments were performed in PBS 1X, pH 7.4 buffer. Black lines, wt MIF; blue lines, L46F MIF; red lines, L46A MIF; green lines: L46G MIF.

Understanding the molecular factors that govern trimer formation and the role of oligomerization in modulating its structural stability and attenuating its biochemical and biological properties is crucial for understanding MIF’s function in health and disease. MIF trimer formation is required for its catalytic activity. The tautomerase active site is formed at the monomer-monomer interface and involves amino acid residues from both neighbouring subunits [42] and the N-terminal catalytic proline residue (Pro1) [40], [42], [43]. Furthermore, studies using recombinant MIF suggest that MIF’s binding to its receptor CD74 involves the trimer [44]. Therefore, small molecules or mutations that inhibit or disrupt trimer formation should allow for simultaneous inhibition of its catalytic activity and receptor binding, thus providing more effective antagonists of MIF’s proinflammatory activity compared to tautomerase inhibitors or neutralizing antibodies. Herein, we describe novel intersubunit interactions involving the hydrophobic packing of leucine 46 (Leu46) side chain on the β-strand β3 of one monomer within a hydrophobic pocket from the adjacent monomer constituted by residues Arg11, Val14, Phe18, Leu19, Val39, His40, Val41, Val42, and Pro43 (**Figure 1**). Analysis of MIF sequences and high resolution X-ray structures of MIF reveals that the formation of this hydrophobic pocket is highly conserved (>95%) or exhibits highly conservative mutations across mammalian (human), rodent (rat and mice) and amphibian (frog) MIF, suggesting that it may play critical roles in modulating MIF trimerization and stability. Interestingly, in nematode MIF the hydrophobic interaction is replaced by tight electrostatic interactions, where Leu46 is substituted by an arginine, and the hydrophobic/basic residues constituting the pocket, Arg11 Leu19 and His40 are replaced by the acidic residues Asp11, Glu19 and Glu40 respectively (**Figure 1D**). To elucidate the structural significance of these intersubunit interactions and their relative contribution to MIF’s trimerization, structural stability and catalytic activity, we generated three point mutations where leucine 46 (Leu46) was replaced by glycine (L46G), alanine (L46A) and phenylalanine (L46F) (**Figure 2A–D**), and their structural properties and stability, oligomerization state, and catalytic activity were characterized using a battery of biophysical methods and X-ray crystallography. In addition, to further analyze the global structural behavior of wild-type (wt) and mutants huMIF, and to investigate the dynamic properties at the atomic scale, we carried out ∼100 ns molecular dynamic simulations. Our results reveal that Leu46 intersubunit interactions play important role in stabilizing the secondary and tertiary structure of MIF. Nonetheless, sedimentation velocity analytical ultracentrifugation, NMR and X-ray crystallography provide strong evidence that these hydrophobic interactions do not influence the oligomerization state of MIF.

## Materials and Methods

### Materials

Polymerase chain reaction (PCR) and other molecular biology reagents were purchased from Stratagene unless stated otherwise. DNA mini and maxi prep reagents were purchased from Qiagen and oligonucleotide primers from Microsynth. Escherichia coli BL21 DE3 cells, rabbit monoclonal anti-huMIF and goat anti-rabbit ALEXA Fluor 680 were purchased from Invitrogen. Miscellaneous chemicals were from Sigma-Aldrich Chemicals and were of the highest grade commercially available. Isopropyl 1-thio-P-D-galactopyranoside (IPTG) was purchased from Applichem. ^15^N-ammonium chloride was purchased from CIL (Cambridge Isotope Laboratories, Switzerland).

### Site-directed Mutagenesis of Leu46 Mutants

Wt huMIF cloned into the pET11b expression vector was a kind gift from Prof Richard Bucala. L46A, L46G and L46F huMIF mutants were engineered by site directed mutagenesis using mutagenesis kit from Stratagene. All mutants were cloned from huMIF-pETllb by DNA amplification. Polymerase chain reactions (PCR) were performed in a Px2 Thermal Cycler (Catalys AG, a Promega Company). Initial denaturation was for 30 s at 95°C followed by 16 cycles of 30 s at 95°C, 1 min at 55°C, and (1 min/Kb of plasmid length) at 68°C using 2.5 units of Pfu Turbo DNA polymerase (Stratagene). Primers designed were 5′ GTC CCT GAT CAG TTC ATG GCC TTC GGC 3′ (sense), 5′ GCC GAA GGC CAT GAA CTG ATC AGG GAC 3′ (antisense) for L46F huMIF; 5′ GTC CCT GAT CAG GGC ATG GCC TTC GGC 3′ (sense) and 5′ GCC GAA GGC CAT GCC CTG ATC AGG GAC 3′ (antisense) for L46G huMIF; and 5′ GTC CCG GAC CAG GCC ATG GCC TTC GGC 3′ (sense) and 5′ GCC GAA GGC CAT GGC CTG GTC CGG GAC 3′ (antisense) for L46A huMIF. Mutants DNA sequences were confirmed by sequencing at Microsynth. Note that residue numbering throughout this manuscript starts at Pro-1 and not at the cleaved initiator methionine.

### Protein Expression and Purification

Expression and purification of wt and mutants were carried out as described previously [36]. The wt and three mutants are expressed as soluble proteins in *Escherichia coli* (*E.* c*oli*), and the purity and protein identity were verified by sodium dodecyl sulfate polyacrylamide gel electrophoresis (SDS-PAGE) Coomassie blue staining, reversed-phase high performance liquid chromatography (HPLC) and matrix-assisted laser desorption ionization time of flight (MALDI-TOF) mass spectrometry. All proteins were found to be more than 95% pure; mass spectrometry analyses revealed single peaks with the expected average molar masses of 12345 g.mol^−1^, 12303 g.mol^−1^, 12379 g.mol^−1^ and 12289 g.mol^−1^ for wt, L46A, L46F and L46G respectively. For preparation of uniformly ^15^N single-labeled and ^13^C/^15^N-double-labeled huMIF, samples were prepared by growing the bacteria in M9 minimal media containing ^15^N-ammonium chloride (1 g/L) as the only nitrogen source, or ^15^N-ammonium chloride and ^13^C-glucose in case of double labelled samples, supplemented with minerals and cofactors [45].

### MIF Keto-enol Tautomerase Activity

The keto-enol tautomerase activity of MIF was measured using the hydroxyphenylpyruvate substrate as described previously [36], [46]. Total enzyme concentration in the reaction mixture was 100 nM and the catalysis was followed for one minute using the CARY 100 Bio UV-Visible Spectrophotometer at 475 nm. Initial rate of the catalytic activity was calculated within the first 30 s of the reaction. Data calculated are the average of at least three measurements.

### Probing the Structural Stability of MIF by Far-UV Circular Dichroism (CD) and Fluorescence Spectroscopy

The far-UV (195–250 nm) CD spectra of wt and huMIF mutants in PBS 1X **(**137 mM NaCl, 10 mM Phosphate, 2.7 mM KCl, pH 7.4**)** were recorded at room temperature using a 0.1 cm quartz cell and Jasco J-815 CD Spectrometer equipped with a thermostated cell holder. Data were acquired at a step size of 0.5 nm, an averaging time of 0.25 to 2 s, a bandwidth of 1 nm, and an average of five scans recorded to generate the data reported in units of mean molar ellipticity per residue. Thermal denaturation (TD) studies were performed by recording the mean molar ellipticity at 218 nm for each protein (5–30 µM) in PBS 1X as a function of the temperature (20–98°C). Data were collected at 218 nm, using a temperature slope of 2°C/min with data pitch of 0.2°C and a bandwidth of 1 nm. Unfolding curves are expressed as the percentage of unfolded protein relative to native protein (*i.e.* the change in ellipticity at 218 nm) over the increase in temperature. Guanidinium hydrochloride (GdnHCl) induced denaturation studies were performed by recording the mean molar ellipticity per residue as a function of wavelength (195–240 nm) and GdnHCl concentration. The spectra represent the average of at least 3 samples (10 µM, in PBS 1X buffer at room temperature). Data were collected at 218 nm with a time constant of 8 s and a bandwidth of 1 nm. Unfolding curves are expressed as the percentage of unfolded protein relative to native protein (*i.e.* the normalized change in ellipticity at 218 nm) over GdnHCl concentration.

GdnHCl induced denaturation studies were also performed by monitoring changes in the tyrosine and tryptophan fluorescence emission maximum of wt and Leu46 mutants as a function of GdnHCl concentration. The spectra represent the averages of at least 3 scans performed on the protein sample (3 µM) in PBS 1X incubated overnight at room temperature with different concentrations of GdnHCl. The wt and huMIF mutants were excited at 295 nm and 280 nm. Fluorescence emission was acquired over a wavelength range of 290–450 nm using a LS 55 Perkin Elmer Fluorescence Spectrometer. Unfolding curves are expressed as the percentage of unfolded protein relative to native protein (i.e. the change in maximum fluorescence emission intensity as a function of GdnHCl concentration).

### Quaternary Structure Determination by Analytical Ultracentrifugation (AUC) and Light Scattering

Analytical ultracentrifugation experiments were performed on purified and dialyzed MIF samples at 5, 10 and 30 µM in PBS 1X buffer on a Beckman Optima XL-A analytical ultracentrifuge. Sedimentation velocity experiments were carried out at 20°C using 380–400 µL of protein solution. Data were recorded at rotor speeds of 50,000 rpm in continuous mode at 21°C with a step size of 0.003 cm. The experimentally determined partial specific volume of 0.765 mL/mg was used for calculating the molecular weights of wt and mutants huMIF [37]. Given that this value was determined only for wt huMIF and for comparison purposes, the molecular weights of the mutants were also determined using the calculated (using the program SEDNTERP [37]) partial specific volumes of 0.7336 mL/g, 0.7340 mL/g, and 0.7332 mL/g for L46A huMIF_,_ L46F huMIF and L46G huMIF respectively. The sedimentation velocity absorbance profiles were analyzed as a C(s) distribution of the Lamm equation using SEDFIT [47]. To obtain the molecular weights, the molar mass distributions c(M) were obtained by transforming the corresponding c(s) using SEDFIT.

Static light scattering experiments were carried out on purified MIF samples (20–30 µM in PBS 1X) in volumes of 100 µL. All measurements were performed at room temperature on a DAWN HELEOS II Multi-angle light scattering detector (Wyatt Technology Corp, Santa Barbara CA). The system is also equipped with UV (Agilent 1200 VWD) and refractive index (Wyatt Optilab rEX) detectors. Absolute MWs were determined using ASTRA version 5.3 from Wyatt Technologies, using refractive index-based online protein concentration measurement, based on protein dn/dc of 0.185 mL/g.

### NMR Spectroscopy

NMR spectra were acquired at 27°C on Bruker Avance 600 MHz and 700 MHz NMR spectrometers using a triple-resonance cryo-probe equipped with z-axis self-shielded gradient coils. All NMR measurements were done with 300–500 µM sample concentration dissolved in PBS 1X buffer (pH 7.0) with 10% D_2_O. Spectra were processed with TopSpin (Bruker Biospin, Germany) and NMRPipe [48], and visualized and analyzed with Sparky 3.1 [49].

Two-dimensional ^1^H-^15^N heteronuclear single quantum coherence (HSQC) experiments were recorded for wt, L46A, L46F and L46G MIF. Spectral widths were 8389 Hz (9765 Hz for 700 MHz) in the ^1^H dimension and 1581 Hz (1945 Hz for 700 MHz) in the ^15^N dimension. Resonance assignments were previously published for the same buffer system [50]. Mean weighted ^1^H−^15^N chemical shift differences between different MIF variants were calculated according to the relationship Δδ  =  ({(Δδ^1^H)^2^+ [(Δδ^15^N)/5]^2^}^1/2^)/2. Changes were mapped on the crystal structure using PDB entry 1GDO [51] (1.5 Å resolution) and PyMOL.

### Mass Spectrometry

Mass spectrometric analysis of huMIF was performed by matrix-assisted laser desorption ionization MALDI MS using a linear positive ion mode on an ABSciex 4800 (in the EPFL Proteomics Core Facility). The mass spectrometer was calibrated using a mixture of bovine insulin (5734 Da), ubiquitin (8565 Da), and cytochrome c (12361 Da). Sample preparation: after desalting huMIF sample on a StageTip C18 (Proxeon), one volume of sample was mixed with one volume of matrix. Matrix solution consists of 14 mg/mL of sinapinic acid in 50∶50 water:acetonitrile +0.1% trifluoroacetic acid. A two-layer sample preparation has been selected for the MW analysis.

### X-ray Crystallography

MIF mutants (L46A, L46G and L46F) were crystallized using the hanging drop vapor diffusion method. Each of the mutants L46A, L46G and L46F (2.1 mM, 1.2 mM and 3.4 mM, respectively) was mixed with the reservoir solution containing different concentrations of Ammonium sulfate (1.6 to 2.6 M) in 0.1 M Tris (pH 7.5) and 3% isopropanol. Plates were incubated at 18°C and crystals were formed within 30 min to several hours. For data collection, crystals were flash-frozen in liquid nitrogen after being placed in a cryo-protectant containing 25% PEG 400. Data were collected at the Swiss Light Source (SLS, PXI & PXIII). Data were processed with XDS [52]. The mutants crystals belonged to the P 21 21 21 space group, with three molecules per asymmetric unit.

The structures of MIF mutants were solved by molecular replacement using previously published MIF structure (PDB code 1GD0) as template [51]. Refinement was carried out using REFMAC5 [53], part of the CCP4i program suite [54]. Manual adjustments of the model were made in COOT [55]. Coordinates and structure factors for the L46A, L46G and L46F structures have been deposited in the Protein Data Bank (accession code 4EVG, 4ETG and 4EUI, respectively). The structure of wt huMIF with PDB code 3DJH was selected for our comparative analysis because of its high resolution (1.25 Å).

### Computational Studies

Classical Molecular Dynamics (MD) simulations were based on the crystal structure of huMIF (Protein Data Bank code 1GD0) obtained at 1.5 Å resolution [51]; residues corresponding to the His-tag sequence were removed from the PDB file. Four model systems of the trimeric structure of huMIF were considered: wt, L46A, L46F and L46G. The protonation state of the titratable groups were set as in Orita *et al.* [51]. MD simulations were performed using a parallel version of the GROMACS 4 package [56], [57] using the AMBER/parm98 [58] and SPC [59] all-atom force fields for the protein and water, respectively. All systems are subjected to periodic boundary conditions in the three directions of the Cartesian space and the size of the box is 7.61 nm×7.32 nm×7.56 nm. After 2 ns of MD equilibration, 70, 80, 90 and 72 ns of MD simulation for wt huMIF, L46F huMIF, L46A huMIF and L46G huMIF were collected, respectively. Normal conditions (T = 300 K, P = 1 bar) were achieved by coupling the systems with Berendsen thermostat [60] with a coupling constant tau  = 1.0 ps and Berendsen barostat [60] with compressibility of 4.5 10^−10^ bar^−1^ in all three dimensions. Electrostatic interactions were calculated with the Ewald particle mesh method [61]. A 12 Å cutoff for van der Waals interactions was used. Bonds involving hydrogen atoms were constrained using the SHAKE algorithm [62]. All data analysis was done using GROMACS [56], [57] utilities and all molecular images were made with Visual Molecular Dynamics (VMD) [63].

## Results

To elucidate the role of intersubunit interactions involving Leu46 on the structure and stability of MIF, we compared the structural stability, and biophysical properties of wt, L46A, L46F and L46G mutants at the secondary, tertiary and quaternary structure levels.

### Leu46 Mutants Display Similar Secondary Structure but are Structurally Less Stable than wt huMIF

We first probed the effect of mutating Leu46 residue on MIF’s conformation by far UV CD spectroscopy. Similar to the wt protein, all three mutants display a broad spectrum with negative ellipticity between 209 nm and 222 nm consistent with a conserved mixture of α-helix and β-sheet structures (**Figure 2E**). The relative stability of the secondary structure of Leu46 mutants was then assessed by monitoring the protein denaturation during heat-induced unfolding and in presence of chaotropic salts. Thermal unfolding monitored by far UV CD at 218 nm demonstrated that disruption of Leu46 hydrophobic site induces a clear destabilization of MIF structure stability (**Figure 2F**). Wt huMIF unfolds with an apparent Tm value of 78°C at 10 µM, whereas L46F, L46A and L46G huMIFs began to undergo conformational changes at lower temperatures and displayed apparent Tm values of 73°C, 69°C and 61°C respectively at 10 µM. It is noteworthy that L46G huMIF presents a two-step melting curve with inflexion points at 60°C and 72.2°C. L46G aggregates could already be observed at 65°C. Over the protein concentration range of 5 to 30 µM, we observed virtually identical heat denaturation curves and Tm values for each of the wt protein and Leu46 variants respectively (**Figure S1**). Since huMIF aggregates as it unfolds, thermal denaturation of all mutants was irreversible.

To further probe the effect of Leu46 mutations on the structural stability of MIF, we monitored the unfolding of wt, L46G, L46A and L46F by far-UV CD at 218 nm (**Figure 2G**) as a function of GdnHCl. Consistent with the thermal denaturation assays, cooperative unfolding was observed for each huMIF species with the same stability pattern: wt, L46F, L46A and L46G huMIF showed denaturation midpoints, apparent C_m_ values of 1.82±0.02 M, 1.61±0.01 M, 1.40±0.01 M and 1.09±0.05 M, respectively at 10 µM. We then performed GdnHCl unfolding experiments where we determined the stability of MIF at the tertiary structure level by recording the maximum fluorescence emission intensity upon excitation of Tryptophan at 295 nm as a function of GdnHCl (**Figure 2H**). The order of stability observed by fluorescence is consistent with the thermal denaturation and far-UV CD GdnHCl studies: measured unfolding midpoints at 3 µM were 1.43±0.07 M, 1.27±0.02 M, 1.09±0.08 M and 1.14±0.07 M for wt huMIF, L46F huMIF, L46A huMIF and L46G huMIF respectively. Nonetheless, *C*_m_ values measured by fluorescence spectroscopy are fairly lower than those measured by far-UV CD, which can be explained by the fact that the only tryptophan residue of a MIF subunit is located within the C-terminus β-hairpin, which is more accessible and a structurally more flexible region of the protein. All GdnHCl experiments showed that the L46G variant is the least stable mutant and does not follow a two-state unfolding mechanism. Together, these data suggest that interaction of Leu46 from one monomer with the hydrophobic pocket from the adjacent subunit is critical to the structural stability of the trimer.

### Leu46 Mutants are All Trimers

**Figure 3:**
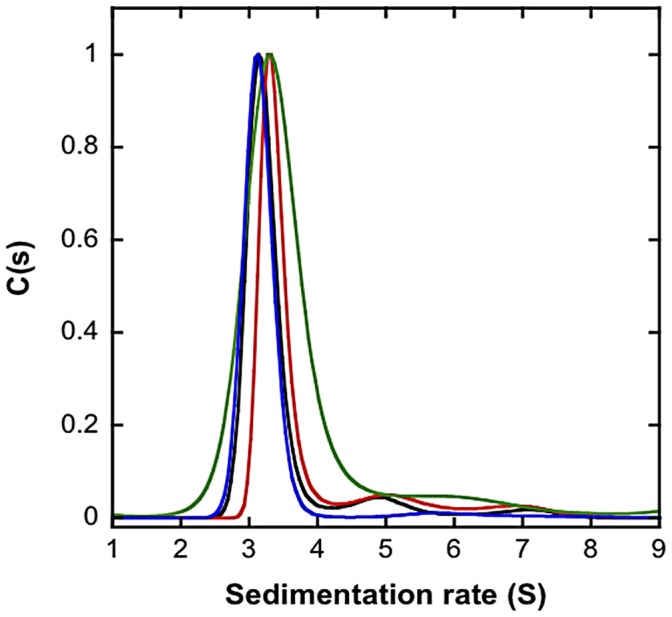
Mutation of Leu46 does not alter the quaternary structure of MIF. Sedimentation rate distributions as determined by Analytical Ultracentrifugation/Sedimentation Velocity experiments indicating similar sedimentation rates for the wt and Leu46 huMIF mutants (15 µM in PBS 1X, pH 7.4 buffer).

Since the Leu46 pocket is located at the monomer-monomer interface and mutating Leu46 destabilizes the trimer, we first sought to determine whether the Leu46 mutations alter the quaternary structure of MIF by analytical ultracentrifugation/sedimentation velocity experiments (**Figure 3**). All huMIF variants sediment predominantly as trimers; wt and L46F exhibit a sedimentation coefficient of 3.15 S while L46A and L46G sediment with an s value of 3.3 S. To determine if the effect of Leu46 mutations on MIF’s oligomerization is concentration dependent, we performed sedimentation velocity studies on all proteins over the concentration range of 5–50 µM. At all concentrations, wt and Leu46 mutants sediment predominantly as a single species with s values and molecular masses corresponding to that of the trimer (**Figure S2**).

To confirm the above results and to examine the consequences of Leu46 mutations on the quaternary structure of huMIF under native conditions, we performed static light scattering studies on wt and mutants at protein concentration of 20 µM. Static light scattering and refractive index detection represent a reliable tool to monitor for structural properties of proteins and determination of their accurate molecular weight. Analyses of our LS data demonstrated that all MIF variants correspond to trimeric structures with MW ∼33 KDa.

### Probing the Importance of Leu46 Hydrophobic Pocket Stability on the Functional Properties of huMIF

Inspired by the proximity of the tautomerase active site and the hydrophobic pocket (**Figure 1B**), we then sought to assess whether destabilization of Leu46 pocket could be transmitted to the catalytic site and affects its conformation. Thus, kinetic parameters of wt huMIF and Leu46 mutants were measured using the hydroxyphenylpyruvate as a substrate (**Table 1**). Our data showed that L46A mutation has almost no effect on MIF’s catalytic activity and affinity towards its substrate (K_m,L46A_ ∼1.04±0.06 mM, K_m,wt_ ∼1.05±0.04 mM; K_cat,L46A_ ∼52.7±2.8 s^−1^, K_cat,wt_ ∼52.9±4.8 s^−1^). The L46G and L46F mutants had opposite, yet small effects on MIF’s enzymatic efficiency and affinity. The L46G mutant exhibits a slightly increased (∼10%) K_m_ value (1.16±0.08 mM) indicating a lower affinity of the mutant to the hydroxyphenylpyruvate. However, catalytic efficiency is unchanged relative to the wt (K_cat_/K_m_ = 48.8 s^−1^.mM^−1^). In contrast, the L46F mutant showed almost 10% increased affinity towards the substrate (K_m_ = 0.94 mM) and a ∼1.5 fold higher catalytic constant (K_cat_ = 81.1 s^−1^), leading to an enhancement of the protein enzymatic efficiency. Our data suggest that Leu46 intersubunit interactions play a role in modulating the catalytic activity of MIF. Perturbing the hydrophobic interactions within the Leu46 pocket, *i.e.* increasing or decreasing the hydrophobic interactions, has different effects on MIF’s catalytic efficiency and affinity towards its substrate (**Table 1**).

**Table 1:** Summarized enzymatic and biophysical data collection of wt and Leu46 huMIF mutants.

	Enzymatic Activity			AUC	Circular Dichroism		Fluorescence	
	Km (mM)	Kcat (s^−1^)	Kcat/Km (s^−1^.mM^−1^)	Sedimentationrate (S)	Tm (°C)	Cm (M)	Cm *Ex.280 nm* (M)	Cm *Ex.295 nm* (M)
Wt	1.05±0.04	52.9±4.8	50.3±4.8	3.13±0.05	78±0.5	1.82±0.01	1.32±0.01	1.43±0.01
L46A	1.04±0.06	52.7±2.8	50.6±4.3	3.28±0.06	69.2±0.5	1.40±0.01	1.08±0.01	1.09±0.01
L46G	1.16±0.08	56.8±7.9	48.8±7.8	3.27±0.05	61±0.5	1.09±0.01	1.00±0.01	1.14±0.01
L46F	0.93±0.02	81.1±7.9	87.2±8.7	3.15±0.04	73±0.5	1.61±0.01	1.23±0.01	1.27±0.01

Each data represented is the average of three independent measurements; apparent Tm values reported are measured at protein concentrations ranging from 5 to 30 µM; apparent Cm values reported by circular dichroism and fluorescence are measured at protein concentration of 10 and 3 µM respectively.

### Structural Characterization by NMR Spectroscopy

**Figure 4:**
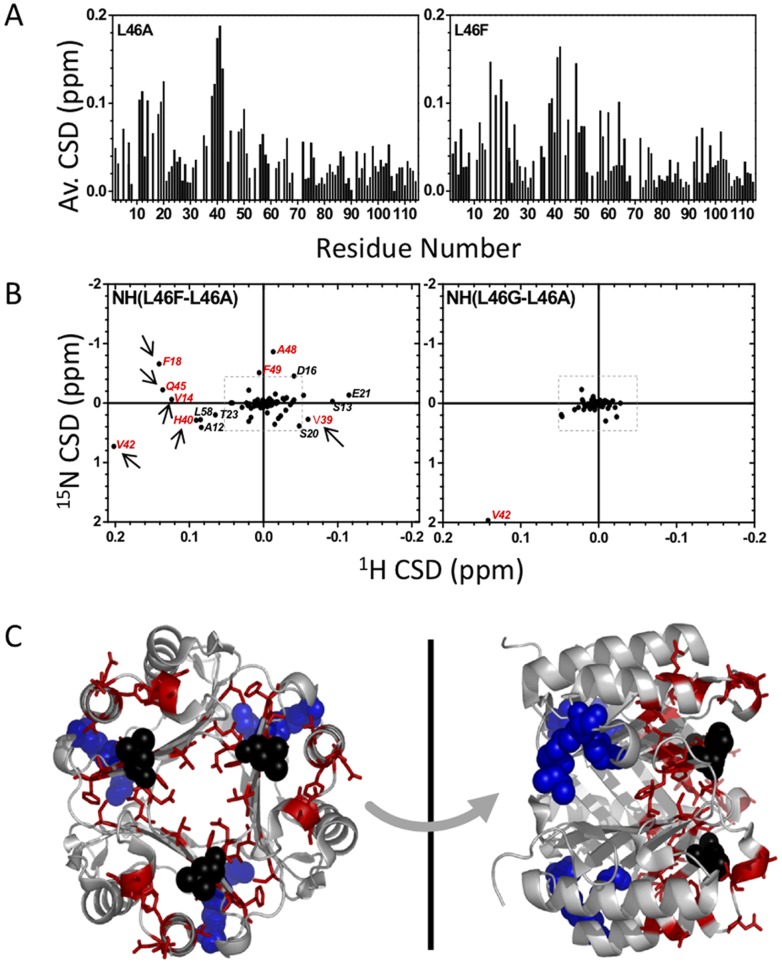
NMR chemical shift perturbations by Leu46 mutants reveal higher fluctuation of the Leu46 pocket. (A) Normalized changes in ^1^H, ^1^5N chemical shifts of L46A and L46F MIF compared to the wild-type protein from wild-type huMIF. Normalized shift changes are calculated according to √(ΔH2+(ΔN/5)2 ). (B) Two-dimensional representation of chemical shift deviations of L46F and L46G MIF from those of the L46A mutant. The gray square is drawn at +/−0.2 ppm in ^15^N, +/−0.02 ppm in the ^1^H dimension and separates very small from larger chemical shift changes. Arrows indicate residues belonging to the hydrophobic pocket. (C) Residues with chemical shift deviations larger than +/−0.2 ppm in ^15^N, +/−0.02 ppm in the ^1^H dimension in L46F and L46A MIF (relative to wt MIF) are mapped onto the MIF crystal structure. Leu46 is shown as black sphere, catalytic core residues (1, 32, 64) as blue sticks, residues with strong chemical shift changes are colored in red.

The effect of disrupting intersubunit hydrophobic interactions, via mutating Leu46, on the structure of MIF, was also assessed using NMR spectroscopy. NMR chemical shifts strongly depend on the chemical environment and are therefore very sensitive to structural changes. **Figure 4** shows chemical shift changes induced by mutation of Leu46 into alanine and phenylalanine. The largest chemical shift changes were observed in the region of residues 1–20 (in particular 12–20) and 38–43 in the case of L46A relative to the wt protein. In the case of L46F, chemical shift changes were observed for residues 12–20 and 39–42, and additional chemical shift changes (compared to L46A) were observed for residues 21–23, 45–49 and residues 58, 60 (**Figure 4B**, left panel and **Figure S3**). L46A and L46G behave very similar with the exception of Val42, which is in direct spatial proximity to the side chain of residue 46 of another monomer (**Figure 4B**, right panel). It is noteworthy that most of the residues having marked dissimilar behavior between L46F and L46A/L46G mutants belong to the hydrophobic pocket; notably: residues Val14, Phe18, Val39, His40, Val42 (**Figure 4B**). For better visualization, residues from L46A and L46F huMIF bearing chemical shift deviations larger than +/−0.2 ppm in ^15^N or +/−0.02 ppm in the ^1^H dimension relatively to wt huMIF are mapped onto the crystal structure of wt huMIF (PDB code 1GD0) (**Figure 4C**).

### Structural Studies by X-ray Crystallography

**Figure 5:**
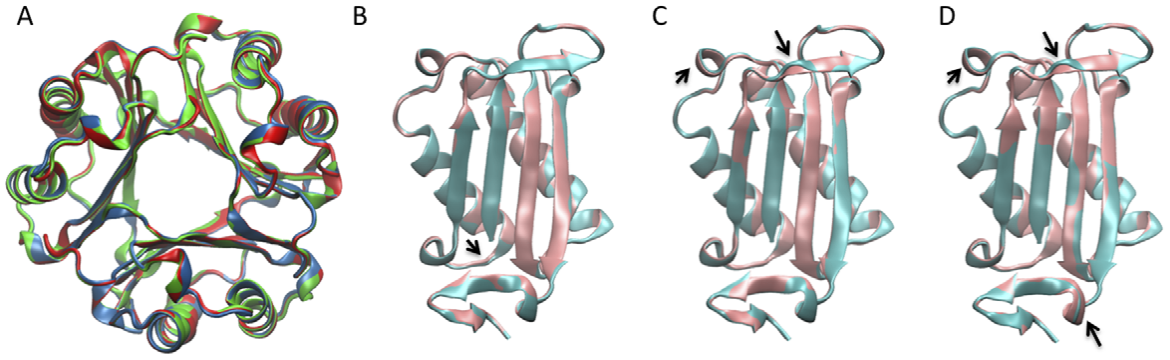
X-ray crystallography demonstrates that the three-dimensional structure of Leu46 mutants is very similar to the wt protein. (A) Overlay of crystal structures of L46F (blue) L46A (green) and L46G (red). (B, C, D) Secondary structure disruptions induced by the Leu46 mutants are shown by superimposition of the wt human and L46F (B), L46A (C) and L46G (D) MIF monomers. Wt and Leu46 mutant monomers are represented in pink and cyan respectively. Black arrows highlight the structural changes induced in the Leu46 variants.

To understand the role of Leu46 mutations on the three-dimensional structure of MIF, we also determined the crystal structures of L46F, L46A and L46G huMIF at 1.70 Å, 1.70 Å and 1.60 Å, respectively. Similar to the wild type species, Leu46 mutants crystallized as homotrimeric proteins with dimensions of approximately 35 Å×50 Å×50 Å. No striking effect to the three-dimensional structure was observed upon mutating Leu46 (**Figure 5**). Root mean square deviations from the initial wt backbone structure are 0.167 Å, 0.574 Å and 1.153 Å for L46F, L46A and L46G respectively. Interestingly, these structural deviations are consistent with the order of stability of the protein observed by circular dichroism and fluorescence. Careful examination of the structure of MIF mutants suggests that substitution of Leu46 by phenylalanine mutation has no significant effect on the secondary structure of MIF but causes a slight distortion of β-strand β3, whereas substitution by alanine (L46A) or glycine (L46G) results in systematic perturbation of the protein’s secondary structure at both the β-strand β3 and the loop located at the N-terminus of α-helix (residues 10–14), in line with the changes in NMR chemical shifts in this region (**Figure 4A** **)**. Additionally, L46G disruption of the hydrophobic pocket induces additional structural changes at the C-terminus where the 3–10 helical structure is lost for a random coil structure (**Figure 5**). The structural effects induced by these mutations correlate with the order of stability of the corresponding mutant protein at the secondary and tertiary structure levels, where the L46G is the least stable mutant and L46F is the most stable variant after the wt (**Figure 2**). Close examination of the structures also revealed slight perturbations of the hydrogen bonds at the interface of adjacent monomers, between β-strands β3 and β2 (**Figure 5** **,** **Table 2**).

**Table 2:** Backbone root mean square deviation of Leu46 mutants based on the structure of wt huMIF, and hydrogen bonds distances stabilizing the internal β-sheet involving β-strands β2 and β3 (Figure 1); percentages represent the increase/decrease of the hydrogen bond distances in the mutants compared to the wt protein.

	Wt huMIF	L46F huMIF	L46A huMIF	L46G huMIF
**RMSD (backbone)**		0.163 Å	0.574 Å	1.153 Å
**G50:N I37:O**	2.806 Å	2.831 Å (+0.9%)	2.856 Å (+1.8%)	2.774 Å (−1.1%)
**A48:O V39:N**	2.902 Å	2.905 Å (+0.1%)	2.943 Å (+1.4%)	2.889 Å (−0.4%)
**A48:N V39:O**	2.892 Å	2.933 Å (+1.4%)	2.869 Å (−0.8%)	2.842 Å (−1.7%)
**x46:O V41:N**	2.991 Å	3.125 Å (+4.4%)	3.052 Å (+2.0%)	2.949 Å (−1.4%)
**Q45:OE1 H40:NE2**	2.865 Å	2.892 Å (+0.9%)	-	2.808 Å (−1.9%)

### Protein Conformational Fluctuations at the Leu46 Hydrophobic Pocket

To understand the dynamic properties of wt and Leu46 huMIF mutants, as well as to better elucidate the molecular basis underlying the effect of the Leu46 mutations, we carried out four independent ∼0.1 µs MD simulations on wt huMIF, L46F huMIF, L46A huMIF and L46G huMIF, based on the crystal structure of the wt protein (PDB code: 1GD0, *see* Method section). Although the limited timescales of MD simulations (of the order of ∼µs) do not capture large conformational rearrangements which often involve timescales of ≥ ms, they can provide some insights about the structural behavior of proteins in solution, at the atomistic level.

### Protein Conformational Fluctuations at the Leu46 Hydrophobic Pocket

The equilibrium states of the wt and Leu46 mutants, obtained upon ∼7 ns of MD equilibration do not largely differ from the initial crystal structure (wt huMIF), based on the root mean square displacements (RMSD). At equilibrium, the RMSD of the Cα atoms fluctuates around an average value of ∼1.5 Å with respect to the X-ray structure (*data not shown*). On the other hand, the root mean-square fluctuations (RMSF) of the residues in wt and Leu46 mutants are virtually similar, indicating that the overall fold of the protein is well maintained (**Figure S4**). Nonetheless, wt huMIF and all of Leu46 mutants show higher fluctuations in the region between residues 13 and 18 (RMSF >1.5 Å), which are located at the N-terminus of the α-helix H1 and participate in the formation of the hydrophobic pocket (**Figure 1**), suggesting a higher mobility of this protein region compared to the rest of the protein. In addition, residues 28–32 of L46F MIF, corresponding to the C-terminus of the α-helix H1, exhibit larger mobility compared to the other proteins, suggesting an accumulation of mechanical strain due to higher steric repulsion upon mutating the Leu46 to phenylalanine. The increased flexibility in this region is in agreement with NMR spin relaxation measurements, which showed that the residue stretches 17–22, 31–33, 51, 52, 55 and 72–75 experience internal motions on the nanosecond timescale [50]. In addition, conformational exchange contributions were observed by NMR spectroscopy for residues 62, 63 and 67, which are close to the catalytic site.

**Figure 6:**
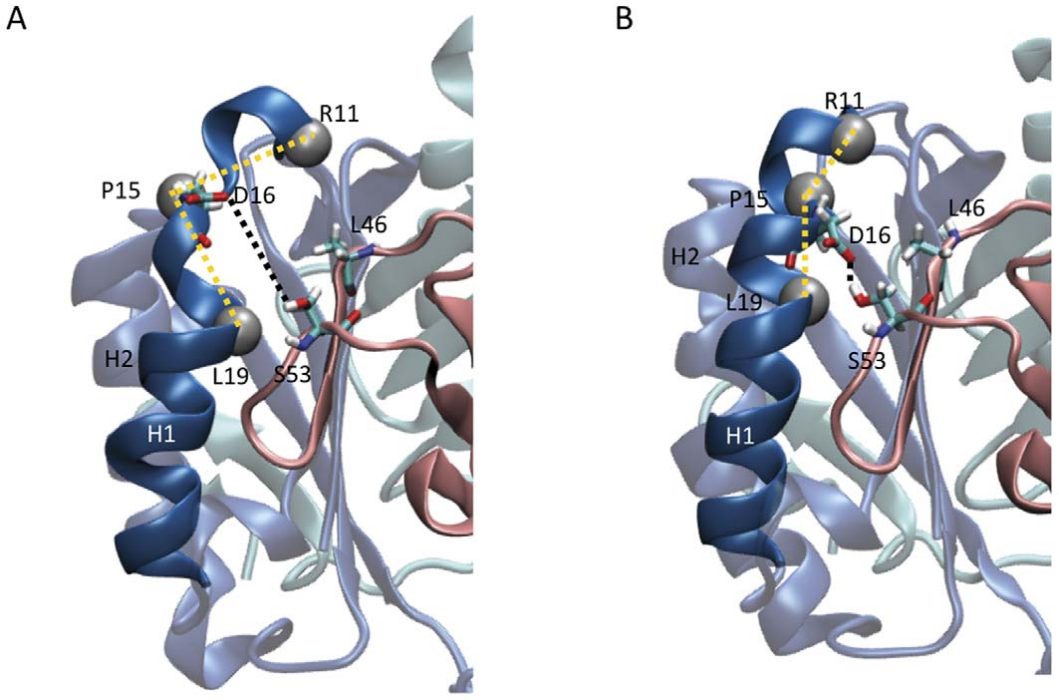
α-helix H1 fluctuates between two states as observed during MD simulation. (A, B) Snapshots of the protein where the angle between the Cα atoms of residues Leu19_Cα_:Pro15_Cα_:Arg11_Cα_ is ∼90° (shortened α-helix H1) and ∼120° (extended α-helix H1), respectively.

Analysis of the MD simulations trajectory of the wt huMIF suggests that the fluctuation of the hydrophobic pocket corresponds to the oscillating motion of the α-helix H1 between shortened and extended states (**Figure 6A, B**). Two different parameters were used to measure the changes in the conformational properties of helix H1; 1) the angle between the Cα atoms of residues Leu19_Cα_:Pro15_Cα_:Arg11_Cα_ (**Figure 6**), located at the N-terminus of H1, which provides a measure of the Leu46 pocket enlargement and fluctuates between two distinct values, ∼90° (shortened α-helix H1) and ∼120° (extended α-helix H1) (**Figure 6A, B**); and 2) the hydrogen bond formation between the polar H of residue Ser53 side chain from the extended α-helix, and the carbonyl of residue Asp16 from the adjacent monomer (distance fluctuates between ∼2 and ∼10 Å) (**Figure 6A, B**). As expected by observing the protein motion, the hydrophobic pocket angle fluctuation is in perfect correlation with the hydrogen bond formation, thus with the extension and shortening of helix H1. When the hydrogen bond Ser53_OH_-Asp_CO_ is formed, the angle Leu19_Cα_:Pro15_Cα_:Arg11_Cα_ is 120° and the α-helix H1 is extended; on the other hand, H1 is shortened when the hydrogen bond is broken, and the angle is 90°. Extension and shortening of the α-helix H1 coupled with the hydrophobic pocket angle fluctuations were also observed for the three Leu46 mutants: L46A, L46G and L46F. This peculiar motion of the α-helix in solution is in agreement with the increased dynamics observed for residues 17–22 by NMR spectroscopy [50]. In its crystal structure, wt huMIF appears with a shortened state of the α-helix H1 [35]. Interestingly, Richardson *et al.* recently reported that MIF’s *Leishmania* homologues, *Leishmania* Major MIF1 (*LmjMIF1*) and *Leishmania* Major MIF2 (*LmjMIF2*) adopt extended α-helix H1 [64] (**Figure S5**).

## Discussion

Several lines of evidence support the importance of MIF’s structure and catalytic activity in regulating some of its biochemical and cellular functions. X-ray crystallography and NMR studies have consistently revealed that MIF exists as stable non-covalent homotrimer, although such studies were done at high, unphysiological concentrations. Nevertheless, the molecular determinants governing the oligomerization of MIF, as well as the specificity of interaction between the different monomers in solution are still being refined. Understanding these factors and the relationship between the structure and functions of MIF are essential for elucidating the molecular basis underlying its multifunctional properties and developing targeted interventions for prevention and therapy of MIF associated diseases. Our approach was first to analyze the interface of MIF subunits and to characterize the residues and key interactions contributing to the specificity and stability of interaction between the different subunits of the trimer.

### Two Types of Interactions Stabilize the MIF Trimer

First, the extensive hydrophobic interface significantly contributes to the affinity between the different monomers, as well as to the stability of the protein tertiary structure. Second, two main regions (C-terminal β-hairpin and β-strand β3, **Figure 1A**) within each monomer participate in several intersubunit polar and hydrophobic interactions. The monomeric form of MIF is unstable and mutations that disrupt MIF inter-monomer contacts lead to misfolding and aggregation of the protein (Farah El-Turk PhD thesis, EPFL) [36], [65]. Therefore, it has not been possible to design a monomeric variant of MIF or develop conditions to populate the monomer in solution. The C-terminal β-hairpin has been demonstrated to play an important role in the tertiary structure and structural stability of the trimer, but is not essential for trimer formation [36]. In the present report, we probed the hydrophobic pocket, located at the N-terminus of the α-helix H1 (**Figure 1A**), where the hydrophobic side chain of Leu46 from the adjacent monomer is packed. This hydrophobic pocket is highly conserved across MIF species (**Figure 1D**), suggesting it might play an important role in stabilizing MIF intersubunit contacts and modulating the structural and functional properties of the trimer. In order to study its relevance to the structure and function of MIF, we used single site-directed mutagenesis to perturb the hydrophobic contacts within the non-polar pocket. Structural and functional properties of wt huMIF, as well as L46F, L46A and L46G mutants were then analyzed and compared using a wide range of biophysical, biochemical and computational techniques. Our study supports a structural model of MIF where the Leu46 pocket plays a role in modulating the structural stability and tertiary structure of MIF.

### Leu46 Hydrophobic Pocket Contributes to the Structural Stability of MIF

As a first step towards understanding the contribution of Leu46 hydrophobic pocket towards MIF trimer stability, GdnHCl titration and thermal melting studies (monitored by far-UV circular dichroism and fluorescence techniques) demonstrated that Leu46 mutants are structurally less stable than the wt protein (**Figure 2**). Nevertheless, all three Leu46 mutants form stable trimers (**Figure 3**). Replacing Leu46 by glycine has the most dramatic effect on protein stability. This could be due to the fact that replacing leucine by glycine results in the exposure of the hydrophobic surface within the pocket, in addition to the increased conformational freedom of glycine. These findings demonstrate that Leu46 side chain interaction within a hydrophobic pocket from the adjacent monomer is a key component of MIF intersubunit interactions.

### Similar to the wt Protein, Leu46 Mutants Exist as Stable Trimers

There are three possible explanations for the decrease in MIF stability upon Leu46 mutation and destabilization of the hydrophobic pocket: (a) dissociation of the trimer and subsequent aggregation of unstable monomeric species, (b) alteration of MIF’s conformation/tertiary structure or changes in the intrinsic flexibility of the protein, (c) changes in the intrinsic flexibility of the catalytic site [36]. To test whether Leu46 mutations alter MIF’s quaternary structure, we determined the oligomeric state of wt and Leu46 mutations by analytical ultracentrifugation/sedimentation velocity and static light scattering. All four proteins (wt, L46F, L46A and L46G) sedimented as a single species corresponding to the trimer. Static light scattering, in agreement with the analytical ultracentrifugation data, demonstrated that wt, L46F, L46A and L46G exist as stable trimers. Finally, crystallographic data and enzymatic assays do not show any drastic conformational change of the catalytic site or activity of MIF upon Leu46 mutations. Therefore, any decrease in the stability of MIF observed by circular dichroism and fluorescence may only be attributed to the alteration in the stability or dynamic properties of MIF’s tertiary and secondary structure.

### Leu46 Hydrophobic Interaction is Essential to the Secondary and Tertiary Structure Integrity of MIF

To test this hypothesis and to probe the influence of the Leu46 hydrophobic pocket destabilization on the structure of MIF, the secondary and tertiary structural properties of the wt and mutants MIF were investigated using NMR spectroscopy. The 2D ^1^H-^15^N HSQC of wt and Leu46 mutant proteins resemble previously observed NMR spectra [50], [66] (**Figure 4**, **Figure S3**), which is in concordance with our circular dichroism and oligomeric studies data (**Figure 2**, **3**). NMR chemical shifts are highly sensitive to the chemical environment and provide excellent probes for the secondary and tertiary structure of proteins. In our case, chemical shift measurements demonstrate an extremely high similarity between L46A and L46G huMIF, whereas the L46F mutant exhibits a slightly different chemical shift pattern.

In agreement with our previous structural studies, X-ray crystal structures of MIF variants demonstrate that the Leu46 mutants conserve the same three-dimensional structure pattern as the wt protein. Nonetheless, our NMR and crystallographic data provide some insight into the possible structural basis underling the proteins stability differences at the secondary and tertiary structure levels, obtained by circular dichroism and fluorescence. First, based on the ^15^N chemical shift deviation plot, residues showing higher conformational deviations have higher secondary and tertiary structure fluctuation in the crystal structures (**Figure 4A** **and** **Figure 5**). Particularly, the residues close to residue 46 (β3) and the hydrophobic pocket show more pronounced changes in NMR signal position (**Figure 4A**). Second, while the L46G mutant has the most drastic effect on the protein secondary structure, the L46F mutation destabilization is slightly observed at the β-strand β3 and at the C-terminus of the α-helix H1 due to an accumulation of mechanical strain caused by higher steric repulsion upon mutating the Leu46 to phenylalanine (**Figure 5B** **)**. Thus, decreasing the hydrophobicity of residue 46 (via mutations L46A and L46G) destabilizes the hydrophobic pocket and results in distant structural perturbation that is transmitted through the backbone, affecting the tertiary structure of the MIF. Henceforth, the hydrophobic interactions at the Leu46 pocket appear to play important role for the conformational properties and stability of MIF.

### MD Simulations Reveal High Level of Fluctuations at the Leu46 Pocket

To further investigate the dynamic properties of the wt and Leu46 MIF mutants, we carried out 100 ns MD simulations on the wt, L46F, L46A, and L46G huMIF. Previous NMR experiments performed by other groups had demonstrated that the N-terminus of α-helix H1 (where Leu46 hydrophobic pocket is located) is a highly fluctuating region compared to other parts of wt huMIF: residues located at the N-terminus of H1 exhibit internal motions on the 1–3 ns timescale [50]. In concordance with these findings, our MD simulations showed that this same region exhibits a high flexibility (**Figure S4**). These data imply that the structural fluctuations within the Leu46 hydrophobic pocket could be involved in the regulation of MIF structure and possibly its function. A notable point about α-helix H1 is the existence of Pro15 within the four N-terminal residues. Proline residue, with very few exceptions, is located in the N-terminus of α-helices, and act as a structural disrupting or switching element of the helix [33]. In our case, MD simulations suggest that the Leu46 hydrophobic pocket fluctuations are caused by shortening and extension of the α-helix H1 due to Pro15, which acts as a switching element (**Figure 6**). Interestingly, MIFs crystallised from different species, where both Leu46 hydrophobic site and Pro15 are conserved, (**Figure 1**) have shortened α-helix H1. Only *Leishmania* proteins (LmjMIF1 and LmjMIF2), where Lys15 substitutes Pro15, crystallize with an extended H1. Richardson and co-workers [64] reported structural differences between *Leishmania* MIF species 1 and 2 and mouse MIF. Parasitic MIFs appear to differ in two key regions from other MIF structures [64]. The α-helix H1 constitutes one of these regions, which extends from residues 13 to 31 in both LmjMIF structures but is significantly shorter in mouse and other species structures (**Figure S5**).

### Fluctuation of Leu46 Pocket Modulates the Conformation of the Enzymatic Pocket

Using hydroxyphenylpyruvate as a substrate, we showed that all Leu46 mutants are enzymatically active. Nonetheless, measurements of the catalytic constants revealed that Leu46 mutations exhibit different effects on MIF’s catalytic activity (**Table 1**). The L46A mutant exhibits very similar catalytic activity and affinity towards the substrate as wt huMIF. L46G yields a slight decrease in huMIF affinity towards hydroxyphenylpyruvate, while L46F mutation leads to increase in MIF’s affinity and catalytic activity. Our data prove that stability of the Leu46 pocket is necessary for the enzymatic activity of the protein. To rationalize the enzymatic data obtained, we sought to acquire structural insights from NMR spectroscopy. Our NMR results demonstrate that the chemical environment at the enzymatic pocket is changed upon mutating the residue Leu46 (**Figure 4**). The structural changes of α-helix H1 are transmitted to residues 35–37 (C-terminus of H1) (**Figure 6**), and trigger a geometric rearrangement of the enzymatic pocket.

### Implications for MIF’s Biological Activity

Despite the fact that we did not investigate the role of Leu46 hydrophobic chamber in regulating MIF’s biological activities, it is important to note that previous studies have suggested that residues involved in formation of the pocket as being essential to MIF’ functions in CXCR2 mediated inflammatory and atherogenic leukocyte recruitment [67]. Weber *et al.* reported about a *pseudo*-(E)LR domain, that is located within the Leu46 chamber, and suggested that this domain is crucial to MIF’s binding to CXCR2. Substitutions of Arg11 and Asp44 by single (R11A) and double mutations (R11A/D44A) severely abrogate CXCR2-mediated functions of MIF in leukocyte recruitment in various *in vitro*, *ex vivo* and *in vivo* models. Arg11 is a crucial component of the Leu46 hydrophobic site: it is located in the vicinity of Leu46 and forms a sort of a “cap” to the pocket (**Figure 1A, D**); Asp44 is located very close to the hydrophobic pocket, as it is adjacent to Pro43 that belongs to the pocket (**Figure 1D**). Weber *et al.* also reported that R11A and D44A single and double mutations do not affect the secondary/tertiary structure of the protein, as assessed by circular dichroism measurements; mutants also exhibited identical tautomerase activity towards D-dopachrome methyl ester. However, the R11A mutant showed slightly more conformational stability than the wt huMIF, as reflected by its mid-point of GdnHCl induced unfolding. This suggests that mutation of Arg11 to a more hydrophobic and less flexible residue could further stabilize the hydrophobic pocket and therefore the entire protein.

### Conclusions

Taken together, our data suggest that the intersubunit interactions involving the residue Leu46 play a key role in the structural stability of MIF and provide new insights into the role of a novel intersubunit hydrophobic pocket in modulating MIF’s conformation, stability, and potentially its receptor binding and biological activity. It is plausible that molecules that effectively compete for the Leu46 pocket and are also large enough to interfere with intersubunit interactions could act as either modulators of MIF activity or as trimer disruptors and more effective drugs for neutralizing MIF *in vivo*.

## Supporting Information

Figure S1 **Thermal denaturation of wt and mutant MIF is not concentration-dependant.** Thermal unfolding studies of wt huMIF (A), L46F huMIF (B), 46A huMIF (C) and L46G huMIF (D) were monitored by far-UV CD at 218 nm. Proteins were prepared in PBS 1X, pH 7.4. Solid lines, 30 µM; dashed lines, 10 µM; dotted lines, 5 µM.
(TIFF)

Figure S2 **Sedimentation rates of wt and Leu46 mutants are independent of protein concentration, in the range tested (5–50 µM).** C(s) distributions of wt huMIF (A), L46F huMIF (B), L46A huMIF (C) and L46G huMIF (d) at 50 µM (solid lines), 15 µM (dashed lines) and 5 µM (dotted lines).
(TIFF)

Figure S3 **NMR chemical shift measurements demonstrate a high similarity between L46A and L46G huMIF, whereas the L46F mutant exhibits a slightly different chemical shift pattern.** (A) Two selected regions in^ 1^H-^15^N HSQC spectra are shown for residues with strong chemical shift deviation. Color codes are as follows: wild-type in black, L46A mutant in red, L46F mutant in green, L46G mutant in blue. (B) Chemical shift differences between L46G MIF and wild-type MIF. (C) Two-dimensional representation of chemical shift deviations of mutant MIF from those of wild-type MIF. The gray square is drawn at +/−0.2 ppm in ^15^N, +/−0.02 ppm in the ^1^H dimension and separates very small from larger chemical shift changes.
(TIFF)

Figure S4 **Root mean square fluctuations (RMSF, a measure of the average atomic mobility) of the Cα atoms during the molecular dynamics simulations of wt and Leu46 mutants.** Black line, wt huMIF; blue line, L46F huMIF; red line, L46A huMIF; green line, L46G huMIF.
(TIFF)

Figure S5 **MIF Leishmania homologues adopt extended α-helix H1.** (A) Superimposition of wt human and *Leishmania* MIF monomers. Note the extension of the helix H1 in the *Leishmania* species, in comparison to the crystal structure of the human protein. (B) Multiple sequence alignment of wt huMIF and the two species of *Leishmania* MIF. Residues highlighted in squares correspond to the hydrophobic pocket, while residues underlined correspond to the tautomerase enzymatic site.
(TIFF)
