# Supplementary figures and images for: Characterization of Molecular Determinants of the Conformational Stability of Macrophage Migration Inhibitory Factor: Leucine 46 Hydrophobic Pocket
Source: PLoS One. 2012 Sep 21;7(9):e45024. doi: 10.1371/journal.pone.0045024 (PMC3448610; doi:10.1371/journal.pone.0045024)

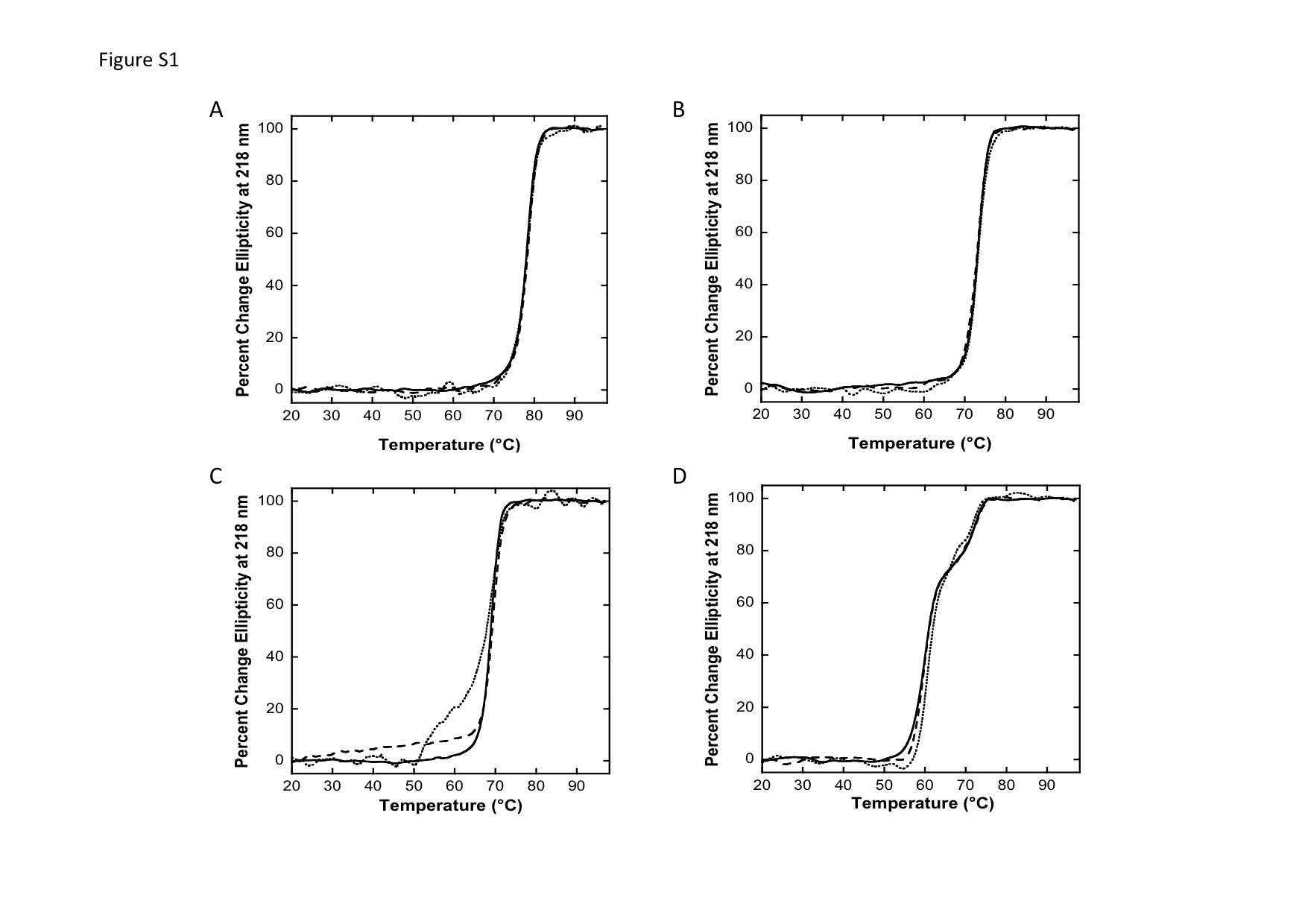

Supplement: Figure S1 — Thermal denaturation of wt and mutant MIF is not concentration-dependant. Thermal unfolding studies of wt huMIF (A), L46F huMIF (B), 46A huMIF (C) and L46G huMIF (D) were monitored by far-UV CD at 218 nm. Proteins were prepared in PBS 1X, pH 7.4. Solid lines, 30 µM; dashed lines, 10 µM; dotted lines, 5 µM. (TIFF) [file pone.0045024.s001.tiff]

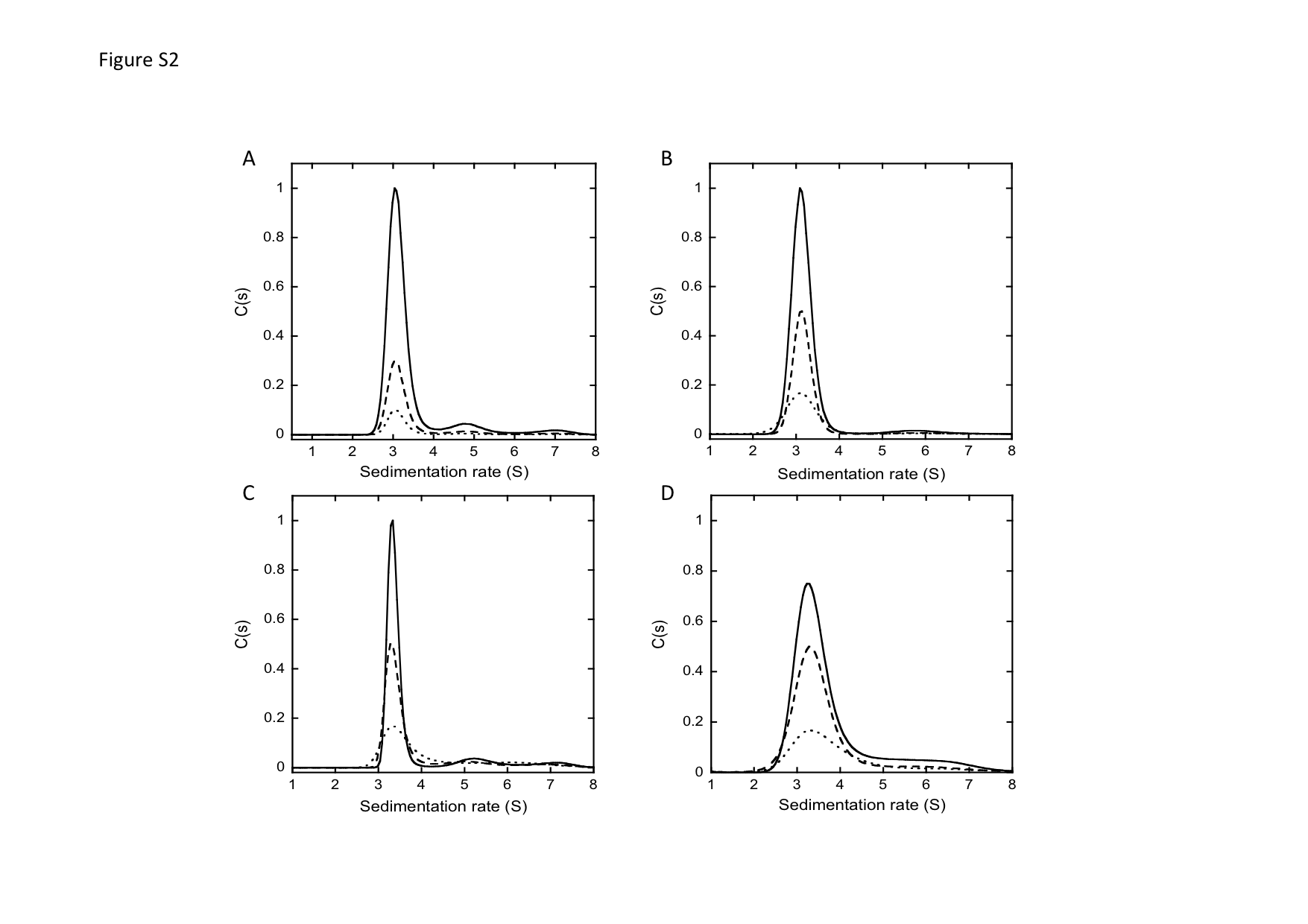

Supplement: Figure S2 — Sedimentation rates of wt and Leu46 mutants are independent of protein concentration, in the range tested (5–50 µM). C(s) distributions of wt huMIF (A), L46F huMIF (B), L46A huMIF (C) and L46G huMIF (d) at 50 µM (solid lines), 15 µM (dashed lines) and 5 µM (dotted lines). (TIFF) [file pone.0045024.s002.tiff]

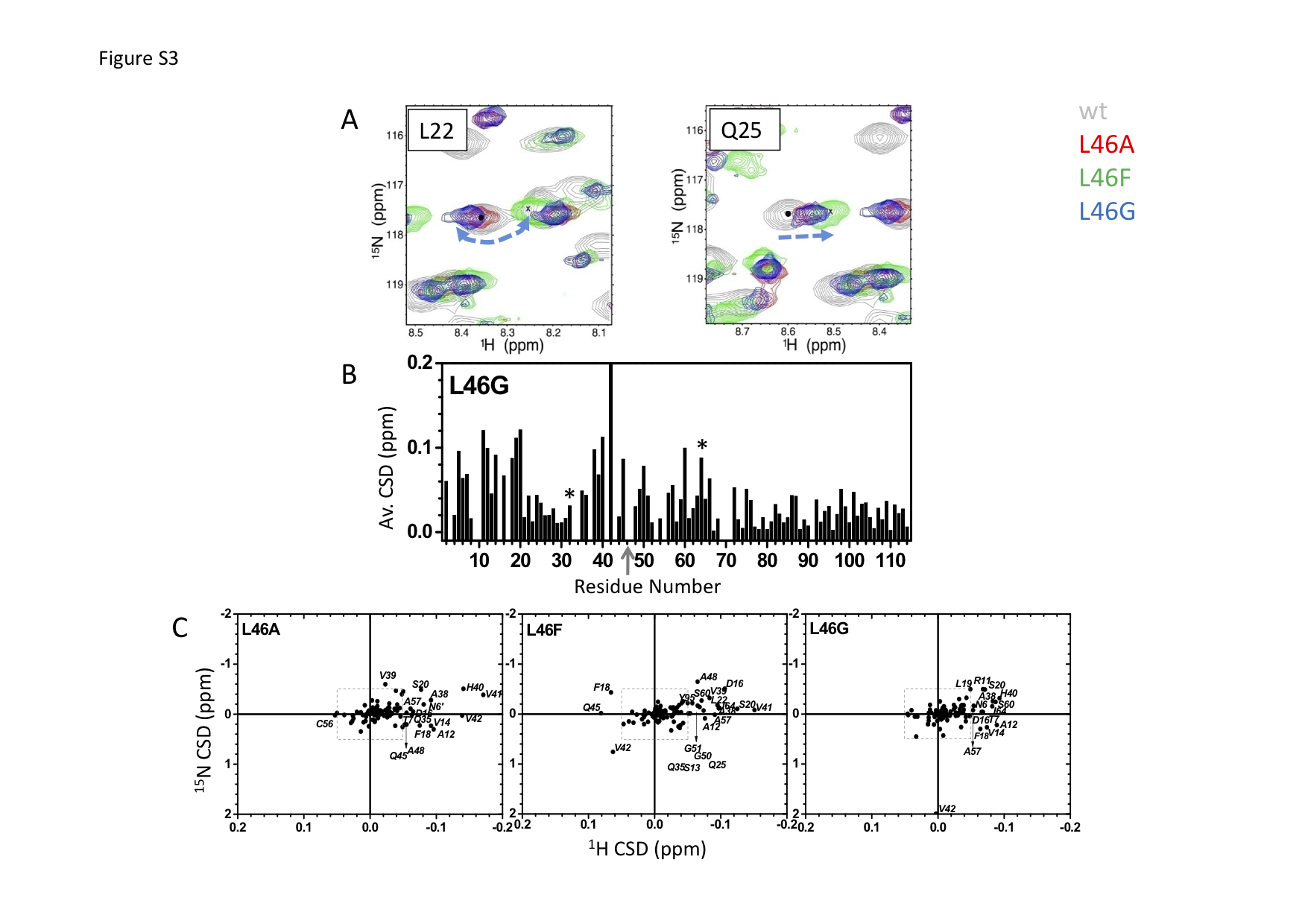

Supplement: Figure S3 — NMR chemical shift measurements demonstrate a high similarity between L46A and L46G huMIF, whereas the L46F mutant exhibits a slightly different chemical shift pattern. (A) Two selected regions in 1H-15N HSQC spectra are shown for residues with strong chemical shift deviation. Color codes are as follows: wild-type in black, L46A mutant in red, L46F mutant in green, L46G mutant in blue. (B) Chemical shift differences between L46G MIF and wild-type MIF. (C) Two-dimensional representation of chemical shift deviations of mutant MIF from those of wild-type MIF. The gray square is drawn at +/−0.2 ppm in 15N, +/−0.02 ppm in the 1H dimension and separates very small from larger chemical shift changes. (TIFF) [file pone.0045024.s003.tiff]

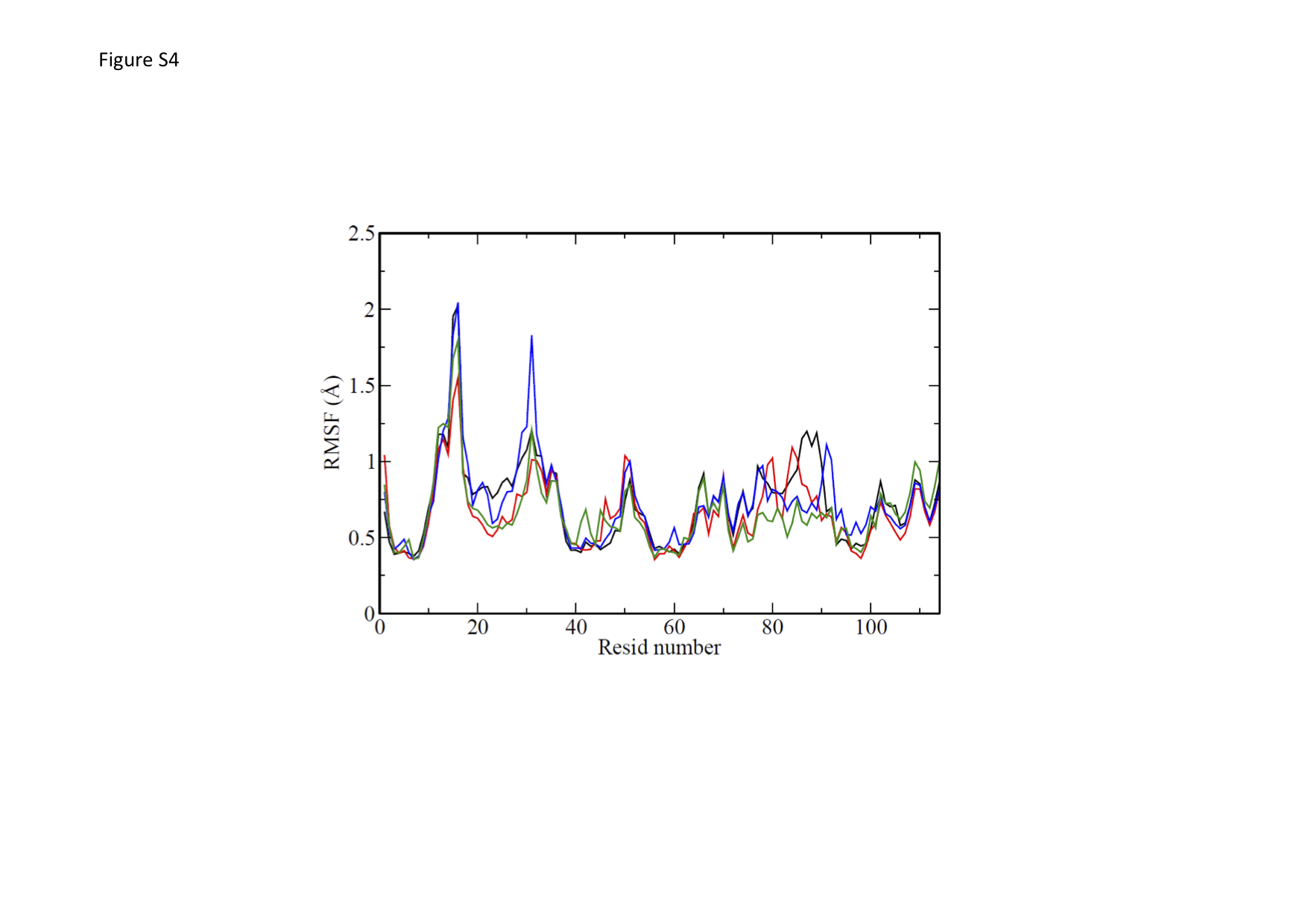

Supplement: Figure S4 — Root mean square fluctuations (RMSF, a measure of the average atomic mobility) of the Cα atoms during the molecular dynamics simulations of wt and Leu46 mutants. Black line, wt huMIF; blue line, L46F huMIF; red line, L46A huMIF; green line, L46G huMIF. (TIFF) [file pone.0045024.s004.tiff]

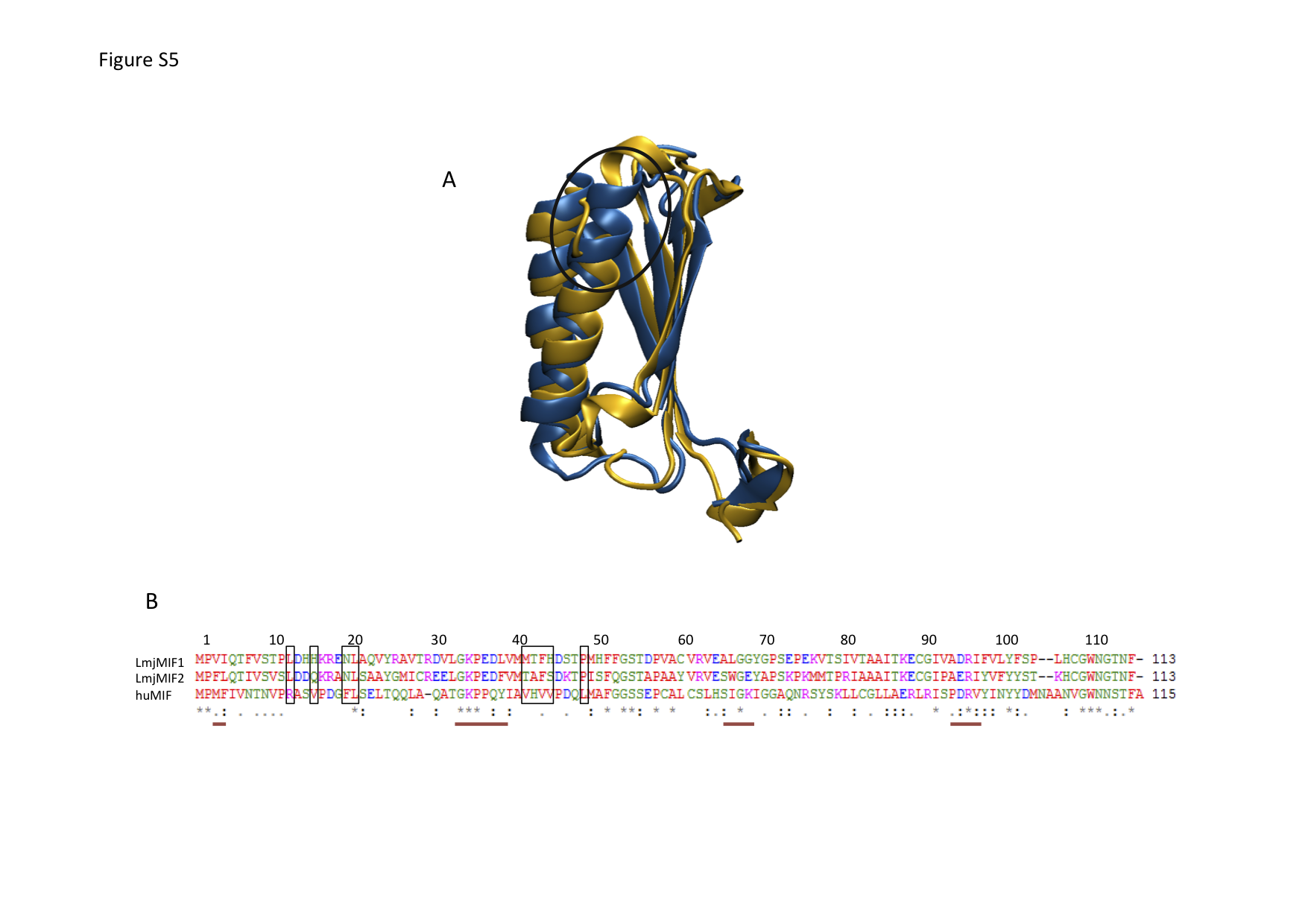

Supplement: Figure S5 — MIF Leishmania homologues adopt extended α-helix H1. (A) Superimposition of wt human and Leishmania MIF monomers. Note the extension of the helix H1 in the Leishmania species, in comparison to the crystal structure of the human protein. (B) Multiple sequence alignment of wt huMIF and the two species of Leishmania MIF. Residues highlighted in squares correspond to the hydrophobic pocket, while residues underlined correspond to the tautomerase enzymatic site. (TIFF) [file pone.0045024.s005.tiff]
